
JOURNAL INFORMATION
==============================
NLM Title Abbreviation: ScientificWorldJournal
Journal ID: ScientificWorldJournal
NLM Journal ID: 101131163
Journal ID: TSWJ

The Scientific World Journal

ISSN: 2356-6140
EISSN: 1537-744X
Publisher: Wiley

ARTICLE INFORMATION
==============================
PMCID: PMC6009329
PMID: 29973777
DOI: 10.1100/tsw.2002.3
Article ID: 235372
Article version: 1
Subjects: Short Report

Structural Genomics: Foundation for the Future of Biology?

Terwilliger Thomas C. *
Bioscience Division, Los Alamos National Laboratory, Los Alamos, NM 87545, USA
*Thomas C. Terwilliger: terwilliger@lanl.gov
Publication date: 2002
Electronic publication date: 2002 Jul 9
Volume: 2
First page: 5
Last page: 6
Copyright: Copyright © 2002 Thomas C. Terwilliger.
Copyright year: 2002
License: This is an open access article distributed under the Creative Commons Attribution License, which permits unrestricted use, distribution, and reproduction in any medium, provided the original work is properly cited.
License URL: https://creativecommons.org/licenses/by/3.0/

Funding: National Institutes of Health

==============================
Advances in Gene Technology: The Genome and Beyond –
Structural Biology for Medicine (Proceedings of the 2002 Miami
*Nature Biotechnology* Winter Symposium)
*TheScientificWorld* 2002, 2(S2), 5–6
ISSN 1532-2246; DOI 10.1100/tsw.2002.3

### STRUCTURAL GENOMICS: FOUNDATION FOR THE FUTURE OF BIOLOGY?

Thomas C. Terwilliger
Bioscience Division, Los Alamos National Laboratory, Los Alamos, NM 87545, and The TB Structural Genomics Consortium (http://www.doe-mbi.ucla.edu/TB)
terwilliger@LANL.gov

**INTRODUCTION**. Structural genomics is the large-scale determination of protein structures. Along with DNA sequencing and functional genomics, structural genomics promises to provide a foundation for developing a fuller understanding of biology. In the short term, structural genomics is likely to be an effective means of obtaining structural information on proteins of high medical, biological, or biophysical interest, including proteins from pathogens or those involved in human disease. *M. tuberculosis* causes TB, a disease that continues to result in over 3 million deaths per year worldwide. Although anti-TB drugs exist, multidrug-resistant strains are becoming increasingly common and the long course of drug therapy discourages proper treatment. Structural genomics provides a promising approach to drug development against this important pathogen.

**METHOD**. The TB Structural Genomics Consortium was formed to target and determine structures of proteins from *M. tuberculosis* on a genomic scale. The Consortium is international in scope and has over 230 members around the world and includes crystallographers, biochemists, geneticists, and TB biologists. The Consortium has targeted over 1000 of the 4000 TB genes and is in the process of carrying out structure determination on those that are amenable to rapid expression, purification, and crystallization or NMR spectroscopy. Those proteins that are more difficult to work with are to be engineered to increase their solubility and expression. The Consortium has policies encouraging collaboration and sharing of data among its members and with the scientific community. The public web site of the Consortium can be viewed at http://www.doe-mbi.ucla.edu/TB.

**RESULTS**. The TB Structural Genomics Consortium has developed technologies for engineering proteins to increase their solubility and for automated structure solution and is applying them to structure determination of TB proteins. The Consortium has a centralized database that is accessible to the public and which shows the targeting and status of structure determinations of proteins from TB. The TB Structural Genomics Consortium welcomes collaborations between its members and any TB biologists interested in structural information. The consortium approach to large-scale structure determination has been effective during the first year of the TB Structural Genomics Consortium, with the structures of 22 proteins from *M. tuberculosis* determined so far by Consortium members.

**ACKNOWLEDGMENT**. This work was supported in part by the National Institutes of Health Protein Structure Initiative.

### REFERENCES

1. Terwilliger, T.C. (2000) Nat. Struct. Biol. 7, 935–939.
2. Waldo, G., Standish, B.M., Berendzen, J., and Terwilliger, T.C. (1999) Nat. Biotechnol. 17, 691–695.
3. Terwilliger, T.C. and Berendzen, J. (1999) Acta Crystallogr. D55, 849–861.
